# Virtual Worlds Technology to Enhance Training for Primary Care Providers in Assessment and Management of Posttraumatic Stress Disorder Using Motivational Interviewing: Pilot Randomized Controlled Trial

**DOI:** 10.2196/42862

**Published:** 2023-08-28

**Authors:** Jennifer K Manuel, Natalie Purcell, Linda Abadjian, Stephanie Cardoos, Matthew Yalch, Coleen Hill, Brittan McCarthy, Daniel Bertenthal, Sarah McGrath, Karen Seal

**Affiliations:** 1 San Francisco Veterans Affairs Health Care System San Francisco, CA United States; 2 Department of Psychiatry and Behavioral Sciences University of California, San Francisco San Francisco, CA United States; 3 Department of Social and Behavioral Sciences University of California, San Francisco San Francisco, CA United States; 4 Department of Psychology Palo Alto University Palo Alto, CA United States; 5 Department of Medicine University of California, San Francisco San Francisco, CA United States

**Keywords:** primary care, posttraumatic stress disorder, PTSD, motivational interviewing, virtual training, training, virtual, stress, disorder, treatment, patient, assessment, communication, feasibility, acceptability, efficacy

## Abstract

**Background:**

Many individuals with posttraumatic stress disorder (PTSD) first present to primary care rather than specialty mental health care. Primary care providers often lack the training required to assess and treat patients with PTSD. Virtual trainings have emerged as a convenient and effective way of training primary care providers in PTSD assessment and communication methods (ie, motivational interviewing [MI]).

**Objective:**

The aim of this study was to conduct a pilot randomized controlled trial of a synchronous Virtual Worlds (VW; a virtual world where learners were immersed as avatars) training versus an asynchronous web-based training on PTSD and MI, comparing the feasibility, acceptability, usability, and preliminary efficacy of 2 different training platforms among primary care providers.

**Methods:**

Participating primary care providers were randomized to a VW and a web-based PTSD training. Outcomes were collected at baseline, posttraining, and 90-days follow-up. Standardized patient interviews measured participants’ communication skills in assessing and managing patients with PTSD symptoms.

**Results:**

Compared to the web-based training, the VW training platform achieved larger learning gains in MI (ie, partnership and empathy) and in discussing pharmacotherapy and psychotherapy for PTSD. Both VW and web-based trainings led to increases in PTSD knowledge and primary care providers’ self-confidence.

**Conclusions:**

The asynchronous web-based PTSD training improved PTSD-related knowledge and self-confidence but was not as effective as the VW immersive experience in teaching MI or clinical management. Because VW training is synchronous and new for many learners, it required more time, facilitation, and technical support. As computer technology improves, VW educational interventions may become more feasible, particularly in teaching clinical skills.

**Trial Registration:**

ClinicalTrials.gov NCT03898271; https://tinyurl.com/mu479es5

## Introduction

Rates of posttraumatic stress disorder (PTSD) among veterans of Iraq and Afghanistan are estimated to be as high as 30% [1,2]. Veterans underuse Department of Veterans Affairs (VA) mental health services due to factors such as poor access, beliefs about psychotherapy, and stigma [3-7]. Veterans more frequently present to VA primary care providers (PCPs) with symptoms of PTSD (eg, sleep disturbance); however, PCPs often fail to associate these symptoms with PTSD. In a prior study [8], PCPs in VA primary care accurately identified PTSD in only half of veterans. In a national survey of PCPs [9], participants answered only 41% of PTSD knowledge questions correctly. Failure to detect and manage PTSD symptoms in primary care patients hinders early intervention and puts patients at risk for chronic PTSD symptoms. Motivational interviewing (MI) is an evidence-based communication method designed to enhance motivation for change [10], including treatment engagement for mental health disorders, such as PTSD.

Given cost constraints, scheduling challenges, geographic barriers, and concern about in-person gatherings (during the COVID-19 pandemic), health care systems increasingly use web-based and virtual reality trainings for continuing medical education (CME) and medical education [11,12]. A meta-analysis of over 200 web-based CME trainings demonstrated large effect sizes in improving self-reported knowledge and clinical practice behaviors, compared to no education [13]. CME programs that included interactive activities and skills practice had the largest effect (*d*=0.67) [14], yet few web-based CME programs achieved high levels of interactivity or immersion.

This appears true both in general and for PTSD training specifically. For example, our team previously developed and piloted an asynchronous, web-based PTSD training for PCPs [15], which included clinical vignettes demonstrating PTSD assessment and management using MI [10]. The results of this study indicated that PCPs’ PTSD knowledge and perceived self-efficacy improved compared to baseline, and PCPs experienced few technical challenges with the web-based training. However, they commented on the lack of interactivity and skills practice [15]. A more engaging platform uses Virtual Worlds (VW), a 3D computer-based multiuser multimedia environment. A VW platform offers graphical representation of a physical space, where individuals use avatars (digital self-representations) to interact with each other and objects [16]. Studies demonstrate that VW training enhances learning outcomes beyond what is provided by web-based or face-to-face learning activities [12,16-18]. However, there is little research on VWs’ potential efficacy for the assessment and management of PTSD.

In this study, we developed a synchronous VW PTSD and MI training for PCPs that was interactive and immersive, simulated trauma and PTSD symptoms, and allowed learners to interact with instructors and each other. The VW also allowed for real-time feedback, as participants practiced new MI and PTSD assessment and management skills with standardized patients (SPs). Using prior asynchronous web-based training with similar content, this pilot randomized controlled trial (NCT03898271) assessed the feasibility, acceptability, usability, and preliminary efficacy of the VW training in improving PTSD-related assessment and management as well as MI skills among PCPs. We hypothesized that those PCPs who participated in the VW PTSD and MI training would demonstrate greater PTSD assessment and MI communication skills, greater self-reported improvements in PTSD assessment and MI communication skills, and greater satisfaction with VW training, compared to participants in the control group who underwent asynchronous web-based training.

## Methods

### Participants, Recruitment, and Randomization

PCPs (ie, physicians, nurse practitioners, or other trainees) from the VA, other community health care systems, and university affiliates across the United States were recruited via email. PCPs with at least five military service veterans on their panels were eligible; PCPs lacking adequate computer or internet speeds to support VW technology were excluded. Following baseline self-report assessments and SP interviews, consenting PCPs were randomized to either the VW training (intervention) or the web-based training (control).

**Ethical Considerations**

The study was approved by the Institutional Review Board of the University of California, San Francisco, and the Research Protection Program of the San Francisco VA Health Care System (IRB number 14-15004). All participants provided consent for their participation in this study. Participants received a gift card valued up to US $50 and the opportunity to earn up to 5.75 CME credits for participation (the renumeration amount varied depending on whether the participant opted to receive CME credit). Participants also received US $20 if they participated in a qualitative interview at the end of the study. All study data have been deidentified in this manuscript.

### Study Overview

The overall goal of this pilot randomized controlled trial was to assess the feasibility, acceptability, usability, and preliminary efficacy of a VW training format, compared to an asynchronous web-based training in improving PTSD-related assessment and management as well as MI skills among PCPs. PTSD assessment skills were measured by participant self-report measures. PCP skills were assessed via standardized behavioral coding and participant self-report measures. The feasibility and acceptability of the training formats (ie, VW and web-based training platforms) were evaluated in qualitative interviews with participants.

### Training Conditions

#### VW Training in PTSD Assessment and MI

The VW training was developed as a collaboration between the research team (content expertise), virtual educational consultants, and a technical build team. The VW training was iteratively refined based on feedback and input from a series of semistructured interviews and focus groups with project stakeholders (ie, PCPs, VA leadership, medical educators, Department of Defense partners, and IT experts). The final VW training consisted of a VW orientation and 2 synchronous 90-minute training sessions 2 weeks apart.

**Figure 1:**
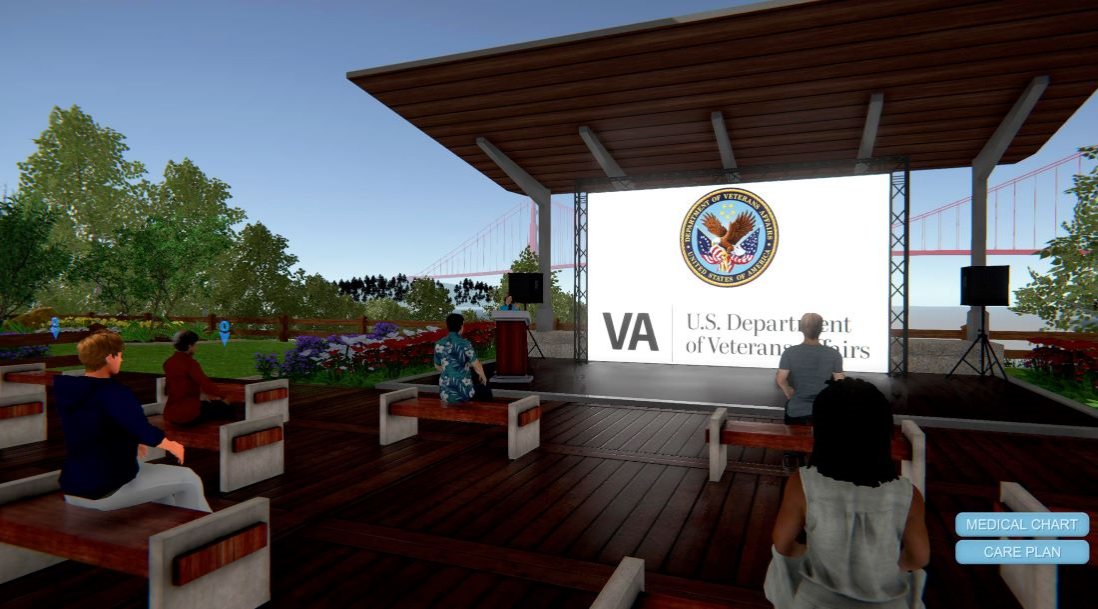
Virtual Worlds screenshot of amphitheater.

**Figure 2:**
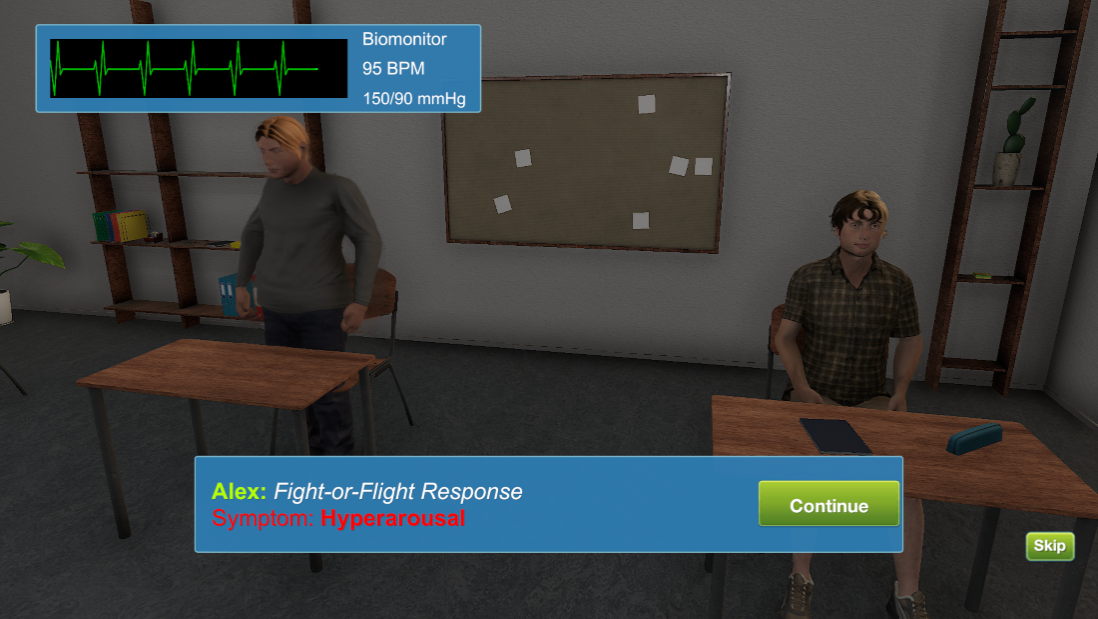
Virtual Worlds screenshot of Alex in classroom.

**Figure 3:**
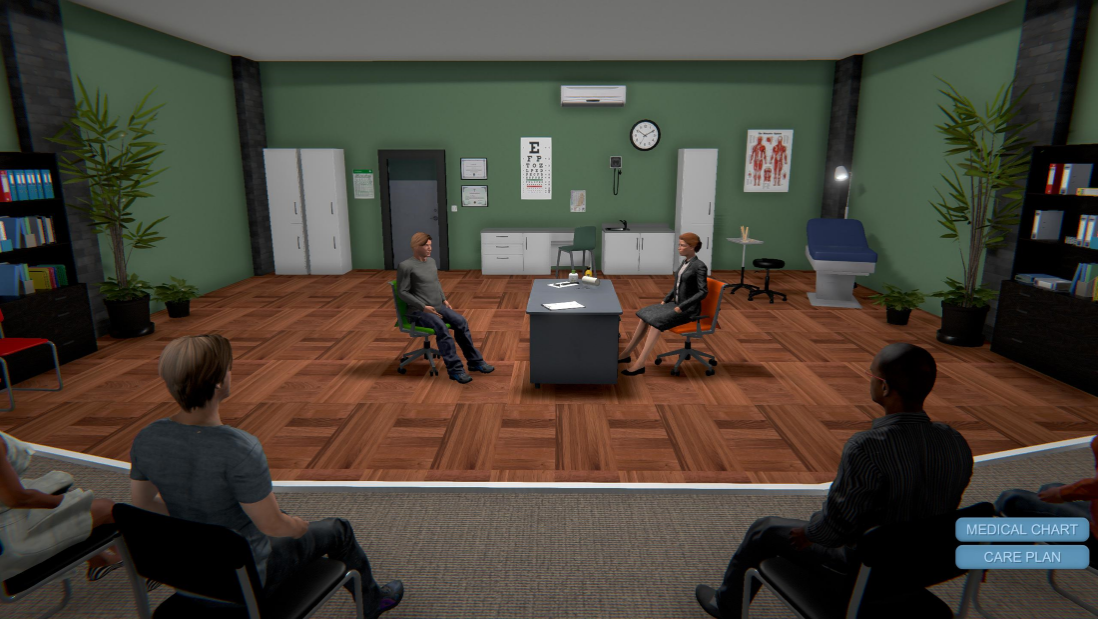
Virtual Worlds screenshot of Alex at the doctor.

**Figure 4:**
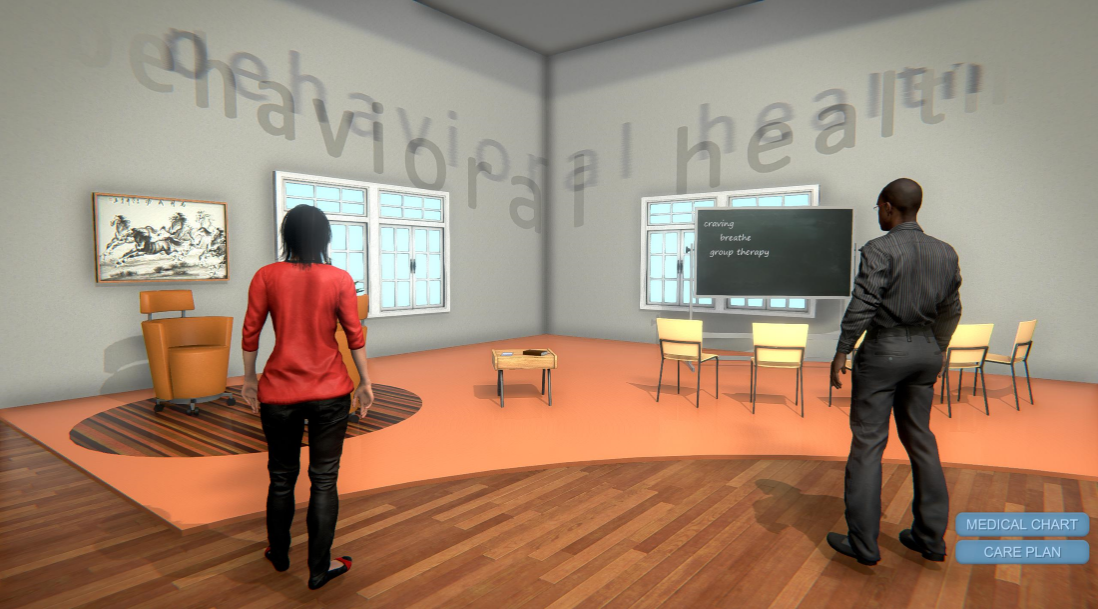
Virtual Worlds screenshot of multimodal care with participants.

Session 1 began in a simulated VA medical center lobby containing informational posters about war-related PTSD. Learners met Alex, a young war veteran with PTSD symptoms who was interviewed by a PCP in the VW environment. Alex only disclosed his symptoms after the VW PCP adopted an MI-consistent communication style. Next, as avatars, learners were virtually teleported to Alex’s apartment, where they assumed Alex’s identity. As “Alex,” participants toured Alex’s apartment for PTSD-related symptoms or “clues” (eg, empty beer bottles, concerned wife, and unused sporting gear). Next, participants were teleported to Alex’s classroom, where they observed his physiological reactivity to innocuous triggers (eg, loud noise) via simulated electronic vital signs monitor. As “Alex,” participants observed how the loud noise triggered Alex’s memory of the battlefield (ie, a flashback of Alex crawling through the battlefield). Next, learners navigated their avatars into an amphitheater for a live, instructor-led didactic session on PTSD symptoms and MI. Finally, learners entered a virtual breakout room where they practiced new MI communication skills by interviewing SPs (also avatars), assessing for PTSD symptoms, and receiving personalized feedback from trained MI experts. Figures 1-4 show screenshots of the VW training.

Session 2 opened with a virtual obstacle course simulating the common barriers to accessing mental health care. From there, participants were teleported to a virtual “Modalities to Care” room, which exhibited 4 multimodal approaches that could be combined to manage Alex’s PTSD symptoms. These approaches included medication, psychotherapy, complementary and integrative health, as well as valued activities. Learners practiced creating SMART (specific, measurable, action-oriented, realistic, and timebound) treatment goals based on patient histories that incorporated a multimodal approach. Learners then entered an amphitheater for a didactic session on PTSD symptom management, including indications for referrals and the use of MI for mental health treatment engagement. The training session again concluded with learners practicing with SPs and receiving feedback on PTSD symptom management and MI skills.

#### Web-Based PTSD Training

The updated asynchronous web-based training (control) was 70 minutes and consisted of an introductory module and 4 web-based narrated video training modules (assessment of PTSD, comorbid conditions and related problems, pharmacological management of PTSD in primary care, and psychotherapeutic interventions for PTSD) [14]. It included didactic content, case presentations, and videotaped clinical vignettes of PCPs interacting with patients with PTSD symptoms using MI communication techniques. Audience polling with questions was added after each module to make the web-based training more comparable to the highly interactive VW training.

### PCP Self-Report Measures

PCP participants were emailed a link to complete the web-based baseline and posttraining self-report measures 1 week and 90 days after training completion. Outcome assessment domains included sociodemographic or clinical practice characteristics (baseline only), PTSD knowledge (8 items) [14], PTSD clinical skills self-confidence, System Usability Scale (SUS) [19-21], and participant feedback on the training.

### SP Interviews and Coding

SPs were trained actors portraying 1of 6 randomly assigned cases of veterans with PTSD symptoms. Participants completed a telephone interview with an SP at baseline, posttraining, and 90-days follow-up. Interviews were coded to evaluate PCPs’ communication and PTSD-related clinical skills in the following domains: (1) shared decision-making and patient engagement, (2) PTSD symptom assessment and symptom management, and (3) MI skills. Each domain included subcategories that were coded on a 5-point global rating scale. The MI domain measured PCP partnership and empathy from the Motivational Interviewing Treatment Integrity behavioral coding system [22]. Other coding domains were developed specifically for this project (Multimedia Appendix 1).

All coders received training and attended weekly coding meetings. SP interviews were deidentified, and coders were blinded to session order (ie, baseline, posttraining, and 90 days). Roughly 20% of interviews were randomly selected for double coding to calculate interrater reliability (IRR).

### Qualitative Interviews With PCP Learners

Qualitative semistructured interviews were conducted with a subset of PCPs to learn more about their experiences with either training platform. Each interview was analyzed by 2 trained analysts using rapid qualitative analysis. Analysts listened to each interview and created a summary of interview content to identify themes with exemplary quotations.

### Data Analysis

Using paired (2-tailed) *t* tests, within-treatment group change in the proportion of correct PTSD knowledge responses were compared from baseline to posttraining and follow-up. A difference-in-differences analysis compared the mean change over time between treatment groups for knowledge or self-confidence and standardized patient coding scores. Coded items were grouped into concepts that measured the same underlying psychometric construct and standard *t* tests compared constructs within study arms. IRR analyses evaluated consistency between coders [23]. Participant feedback was evaluated by comparing mean responses in SUS between groups using standard *t* tests.

## Results

### Demographics and Training Completion

**Figure 5:**
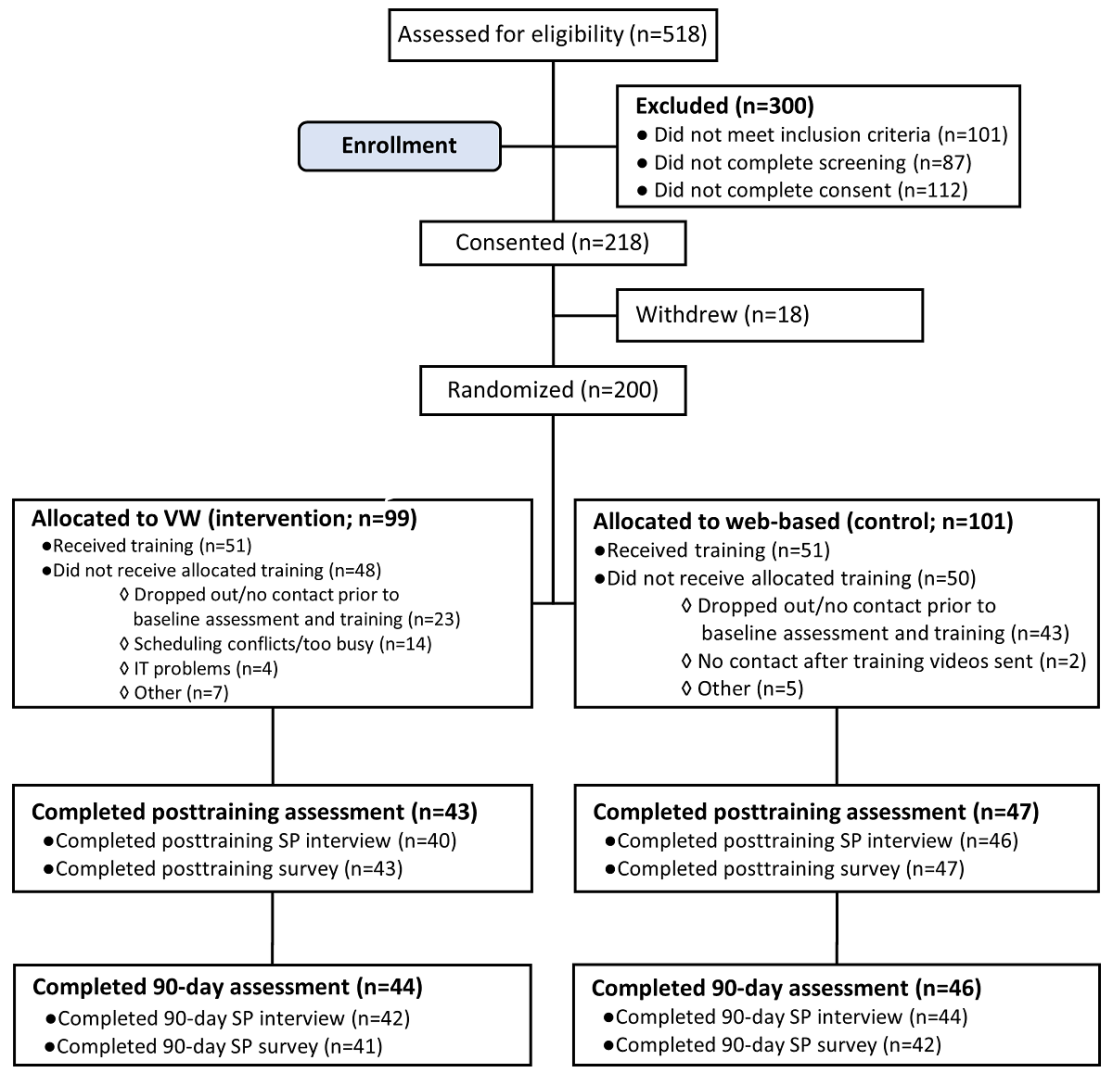
CONSORT (Consolidated Standards of Reporting Trials) diagram of participant enrollment.

Recruitment and enrollment of PCP participants in the trial is shown in Figure 5. Of 200 eligible PCPs, 99 were randomized to the VW (intervention) training and 101 to the web-based PTSD training (control). In the VW condition, a total of 51 participants received training, and 48 did not receive training for various reasons. Specifically, 23 PCPs dropped out or had no contact before the training, 14 dropped out due to a lack of time, 4 had IT barriers, and 7 cited other reasons; Figure 5). In the web-based training, 51 participants received training, and 50 participants did not receive training. Among those who did not receive training, 43 dropped out or had no contact before the training, 2 had no contact after the videos were sent, and 5 cited other reasons for not participating. In sum, a total of 102 PCPs (51 in each arm) completed training. Characteristics of study participants are shown in Table 1.

**Table 1:** Respondent demographic and baseline characteristics by treatment arm (Virtual Worlds [VW] vs web-based training).

Characteristics			Overall (n=107), n (%)		VW training (n=51), n (%)		Web-based training (n=56), n (%)
Gender (female)			72 (68)		32 (64)		40 (71)
**Profession**							
	Physician	57 (53)		31 (61)		26 (46)	
	Nurse practitioner or physician assistant	31 (29)		11 (22)		20 (36)	
	Other or trainee	19 (18)		9 (18)		10 (18)	
**Years since training completed**							
	0-5	23 (22)		9 (18)		14 (25)	
	5-10	17 (16)		10 (20)		7 (13)	
	>10	66 (62)		31 (62)		35 (63)	
Prior VA or DOD^a^ experience			13 (12)		5 (10)		8 (14)
Prior web training experience			86 (81)		40 (80)		46 (82)
**Experience with trauma types^b^**							
	Noncombat trauma	103 (97)		49 (98)		54 (96)	
	Combat trauma	73 (69)		35 (70)		38 (68)	

^a^DOD: Department of Defense.

^b^Not mutually exclusive; no differences between participants in the VW and web-based training conditions were statistically significant.

### Participant Self-Report Items

There was a significant increase in PTSD knowledge post training among participants in both the web-based training condition (Cohen *d*=0.7) and the VW training condition (Cohen *d*=0.6). This increase was maintained at the 90-day follow-up (web-based condition Cohen *d*=0.3 and VW condition Cohen *d*=0.7). Similarly, there was a significant increase in self-confidence in PTSD clinical skills in both the web-based condition (Cohen *d*=1.4) and the VW condition (Cohen *d*=1.2) at posttraining and at the 90-day follow-up (web-based condition Cohen *d*=1.3 and VW condition Cohen *d*=1.5), with no statistically significant difference between groups at either time point (Table 2).

**Table 2:** Percent of PTSD questions correctly answered and mean self-efficacy and self-confidence across the study periods and treatment arms (web-based training vs Virtual Worlds [VW] training).

Metric and arm			Participants^a^, n		Baseline, mean (SD)		Posttraining												
							Mean (SD)		Mean change^b^ difference (95% CI)		*P* value (change difference)		Effect size (95% CI)		DD^c^ (%; 95% CI)		*P* value (DD)		Effect size (95% CI)
**Posttraining PTSD^d^ knowledge**																			
	Web-based	45		72.2 (14.8)		82.2 (12.4)		10.0 (5.0 to 15.0)		<.001		0.7 (0.3 to 1.2)		N/A^e^		N/A		N/A	
	VW	40		71.9 (14.3)		82.1 (17.8)		10.1 (5.3 to 15.0)		<.001		0.6 (0.2 to 1.2)		0.1 (–6.8 to 7.1)		.97		0.0 (–0.4 to 0.4)	
**Posttraining self-confidence**																			
	Web-based	45		62.2 (20.4)		85.6 (10.5)		23.4 (18.8 to 28.0)		<.001		1.4 (1.0 to 1.9)		N/A		N/A		N/A	
	VW	40		55.1 (18.3)		75.6 (15.0)		20.5 (14.6 to 26.4)		<.001		1.2 (0.7 to 1.7)		–2.9 (–10.2 to 4.4)		.43		–0.2 (–0.6 to 0.2)	
**Follow-up PTSD knowledge**																			
	Web-based	39		70.8 (18.6)		76.9 (18.2)		6.2 (0.3 to 12.0)		.04		0.3 (–0.1 to 0.8)		N/A		N/A		N/A	
	VW	38		70.5 (13.7)		80.8 (16.2)		10.3 (5.3 to 15.2)		<.001		0.7 (0.2 to 1.1)		4.1 (–3.4 to 11.6)		.28		0.3 (–0.2 to 0.7)	
**Follow-up self-confidence**																			
	Web-based	39		63.0 (21.1)		84.8 (12.4)		21.8 (16.3 to 27.3)		<.001		1.3 (0.8 to 1.8)		N/A		N/A		N/A	
	VW	38		54.1 (18.5)		78.4 (12.6)		24.3 (19.0 to 29.6)		<.001		1.5 (1.0 to 2.0)		2.5 (–5.1 to 10.0)		.52		0.2 (–0.3 to 0.6)	

^a^Sample size based on the number of participants who completed measures at the respective time points (ie, both baseline and posttraining or both baseline and follow-up).

^b^Mean change from baseline to posttraining or follow-up.

^c^Difference in differences.

^d^PTSD: posttraumatic stress disorder.

^e^N/A: not applicable.

### Participant Self-Report of Training Usability From SUS

Participants in the web-based training reported significantly greater usability (mean 86.9, SD 17.5; Multimedia Appendix 2), compared to participants in the VW group (mean 56.8, SD 21.7), yielding a large effect size (Cohen *d*=1.5).

### SP Interviews

The IRR between coders was moderate for most items (range 0.4-0.7; Table 3). There was a significant within-group increase in the VW group in discussions of pharmacotherapy and psychotherapies from baseline (mean 2.9, SD 0.9) to posttraining (mean 3.4, SD 0.8; Cohen *d*=0.6; Table 4) and follow-up (mean 3.3, SD 1.0; Cohen *d*=0.4; Table 5). There were also significant within-group increases in the domains of partnership and empathy for the VW group from baseline (mean 2.9, SD 0.9) to posttraining (mean 3.3, SD=0.7; Cohen *d*=0.5) and follow-up (mean 3.2, SD 0.8; Cohen *d*=0.4), compared to the web-based group, which remained the same at baseline, posttraining, and follow-up. Between-group differences in partnership and empathy for the VW group, compared to the web-based group, approached significance (*P*=.08; Cohen *d*=0.4) from baseline to posttraining.

**Table 3:** Interrater reliability (IRR) and central tendencies (mean and SD) of coding items.

Metric and items			IRR (95% CI)		Training										
					Baseline				Posttraining				Follow-up		
					Web-based, mean (SD)		VW^a^, mean (SD)		Web-based, mean (SD)		VW, mean (SD)		Web-based, mean (SD)		VW, mean (SD)
**A. Shared decision-making and patient engagement^b^**															
	Overcoming stigma	0.6 (0.4-0.8)		2.5 (0.9)		2.5 (0.9)		2.4 (0.8)		2.6 (0.8)		2.3 (0.6)		2.5 (0.9)	
	Shared decision-making	0.4 (0.2-0.6)		3.6 (1.3)		3.5 (1.2)		3.7 (1.0)		4.1 (1.0)		3.6 (1.2)		4.0 (1.1)	
**B1. PTSD^c^ symptom assessment**															
	Assessment of PTSD symptoms	0.7 (0.5-0.9)		4.5 (0.8)		4.2 (1.1)		4.5 (0.8)		4.37 (0.9)		4.6 (0.7)		4.2 (0.9)	
	Assessment of co-occurring conditions	0.6 (0.33-0.7)		4.3 (0.9)		4.0 (1.0)		4.1 (0.9)		4.03 (0.9)		4.3 (0.8)		4.0 (0.8)	
**B2. PTSD symptom management**															
	Discussion of pharmacotherapy	0.7 (0.5-0.8)		3.1 (1.3)		3.1 (1.4)		3.3 (1.2)		3.5 (1.1)		3.5 (1.2)		3.5 (1.4)	
	Discussion of psychotherapies	0.6 (0.4-0.7)		3.1 (1.3)		2.6 (1.2)		3.3 (1.3)		3.3 (1.1)		3.3 (1.2)		3.1 (1.3)	
**C. Motivational interviewing**															
	Partnership	0.5 (0.3-0.6)		2.75 (1.1)		2.52 (1.1)		2.82 (1.0)		3.24 (1.0)		2.8 (1.0)		3.1 (1.1)	
	Empathy	0.4 (0.2-0.6)		3.15 (1.1)		3.12 (1.0)		3.18 (1.0)		3.50 (0.9)		3.3 (1.0)		3.5 (1.0)	

^a^VW: Virtual Worlds.

^b^All items were rated on a 1-5 scale.

^c^PTSD: posttraumatic stress disorder.

**Table 4:** Change in domain scores in standardized patient assessments from baseline to post-training (web-based training vs Virtual Worlds [VW] training).

Metric and arm		Participant^a^, n		Training																		
				Baseline, mean (SD)		Posttraining																
						Score, mean (SD)		Mean change^b^ (95% CI %)		*P* value (change)		Effect size (95% CI)		DD^c^ (%; 95% CI %)		*P* value (DD)		Effect size (95% CI)				
**A. Overcoming stigma and shared decision-making**																						
	Web-based		44		3.0 (0.9)		3.0 (0.7)		0.0 (–0.3 to 0.3)		>.99		0.0 (–0.4 to 0.4)		N/A^d^		N/A		N/A			
	VW		38		3.1 (0.8)		3.4 (0.7)		0.3 (0.0 to 0.5)		.07		0.3 (–0.1 to 0.8)		0.3 (–0.1 to 0.6)		.19		0.3 (–0.1 to 0.7)			
**B1. PTSD^e^ symptoms and co-occurring conditions**																						
	Web-based		44		4.4 (0.6)		4.3 (0.6)		–0.1 (–0.3 to 0.2)		.58		–0.1 (–0.5 to 0.3)		N/A		N/A		N/A			
	VW		38		4.1 (0.8)		4.2 (0.7)		0.1 (–0.3 to 0.4)		.73		0.1 (–0.4 to 0.5)		0.1 (0.3 to 0.5)		.53		0.1 (–0.3 to 0.6)			
**B2. Discussion of pharmacotherapy and psychotherapy**																						
	Web-based		44		3.2 (1.1)		3.3 (0.9)		0.1 (–0.3 to 0.5)		.59		0.1 (–0.3 to 0.5)		N/A		N/A		N/A			
	VW		38		2.9 (0.9)		3.4 (0.8)		0.5 (0.2 to 0.8)		.002		0.6 (0.1 to 1.0)		0.4 (–0.1 to 0.9)		.12		0.3 (–0.1 to 0.8)			
**C. MI^f^: partnership and empathy**																						
	Web-based		44		3.0 (0.7)		3.0 (0.6)		0.0 (–0.2 to 0.3)		.71		0.1 (–0.4 to 0.5)		N/A		N/A		N/A			
	VW		38		2.9 (0.9)		3.3 (0.7)		0.4 (0.0 to 0.7)		.03		0.5 (0.0 to 0.9)		0.3 (0.0 to 0.7)		.08		0.4 (–0.1 to 0.8)			

^a^Sample size based on the number of participants who completed measures at baseline and posttraining.

^b^Mean change from baseline to posttraining or follow-up.

^c^Difference in differences.

^d^N/A: not applicable.

^e^PTSD: posttraumatic stress disorder.

^f^MI: motivational interviewing.

**Table 5:** Change in domain scores in standardized patient assessments from baseline to post–follow-up (web-based training vs Virtual Worlds [VW] training).

Metric and arm			Participant^a^, n		Training														
					Baseline score, mean (SD)		Follow-up												
							Score, mean (SD)		Mean change^b^ (95% CI)		*P* value (change)		Effect size (95% CI)		DD^c^ (%; 95% CI)		*P* value (DD)		Effect size (95% CI)
**A. Overcoming stigma and shared decision-making**																			
	Web-based	39		3.1 (0.8)		3.0 (0.7)		–0.1 (–0.3 to 0.2)		.55		–0.1 (– 0.5 to 0.4)		N/A^d^		N/A		N/A	
	VW	41		3.0 (0.8)		3.2 (0.8)		0.2 (–0.1 to 0.5)		.16		0.2 (–0.2 to 0.7)		0.3 (–0.1 to 0.6)		.15		0.3 (–0.1 to 0.8)	
**B1. PTSD^e^ symptoms and co-occurring conditions**																			
	Web-based	39		4.3 (0.6)		4.4 (0.6)		0.1 (–0.1 to 0.4)		.27		0.2 (–0.2 to 0.7)		N/A		N/A		N/A	
	VW	41		4.1 (0.8)		4.0 (0.7)		–0.1 (–0.3 to 0.2)		.51		–0.1 (–0.5 to 0.3)		–0.2 (–0.6 to 0.1)		.22		–0.3 (–0.7 to 0.2)	
**B2. Discussion of pharmacotherapy and psychotherapies**																			
	Web-based	39		3.2 (1.1)		3.4 (0.9)		0.2 (–0.2 to 0.6)		.29		0.2 (–0.3 to 0.6)		N/A		N/A		N/A	
	VW	41		2.9 (0.9)		3.3 (1.0)		0.4 (0.0 to 0.8)		.03		0.4 (–0.0 to 0.9)		0.2 (–0.3 to 0.7)		.40		0.2 (–0.2 to 0.6)	
**C. MI^f^: partnership and empathy**																			
	Web-based	39		2.9 (0.7)		3.0 (0.7)		0.1 (–0.2 to 0.3)		.53		0.1 (–0.3 to 0.6)		N/A		N/A		N/A	
	VW	41		2.9 (0.9)		3.2 (0.8)		0.3 (0.0 to 0.7)		.04		0.4 (0.0 to 0.8)		0.3 (–0.1 to 0.7)		.20		0.3 (–0.2 to 0.7)	

^a^Sample size based on number of participants who completed measures at baseline and follow-up.

^b^Mean change from baseline to posttraining or follow-up.

^c^Difference in differences.

^d^N/A: not applicable.

^e^PTSD: posttraumatic stress disorder.

^f^MI: motivational interviewing.

### Qualitative Findings From PCP Learners

PCPs reported mixed perspectives on the value of the VW platform and whether this mode of delivery was worth the time required to install or set up the program and navigate using their avatars. Several participants described the VW as “clumsy” or “inefficient.” Nevertheless, participants overwhelmingly found the content of the VW training memorable and valuable. The interactive and applied components of the training distinguished it from other trainings. Because the VW training incorporated a variety of immersive audio-visual experiences in different virtual settings, participants felt the VW modality was especially strong in accommodating different learning styles. Perspectives were mixed on whether they would choose the VW format again. Some liked the interactive aspects of the VW format, while others liked the greater flexibility and reduced time commitment of the more traditional web-based option. Some participants agreed that any provider who interacts with patients with a history of trauma could benefit from this training, from PCPs to emergency department providers and specialists. Some noted that even though mental health providers are likely to have had training in PTSD, they might not have had as much training in MI and could still benefit from the interactive MI training.

## Discussion

### Principal Results

Results from this pilot randomized controlled trial of a synchronous VW versus an asynchronous web-based training indicate participants in the VW condition achieved greater gains in some dimensions of MI (ie, partnership and empathy) and in their discussions of pharmacotherapy and psychotherapy treatment options with individuals with PTSD. The positive findings regarding the impact of the VW training on MI skills in this study is consistent with other trials of VW training formats to improve PCPs’ MI skills and suggest that this training platform warrants further study [24]. In this study, the web-based training was viewed as more usable compared to the VW format. Nonetheless, both methods of training were successful in increasing PCPs’ knowledge of PTSD assessment and management. These results occurred even though most PCP participants had more than 10 years of professional experience, including experience in caring for patients with trauma. This highlights the importance of ongoing and accessible training in PTSD assessment and management for PCPs.

Data from the SP interviews showed increases in each of the PTSD- and MI-related learning domains measured, many of which were sustained over time. These increases were larger among PCPs who participated in the VW compared to those in the web-based training, but the difference between groups was not significant. Notably, PCPs participating in the VW training achieved significantly higher and sustained MI scores of partnership and empathy. Thus, the VW immersive and interactive activities and practice as well as the real-time feedback may have yielded significantly higher skill levels in engaging patients through empathy and partnership and in discussions about PTSD symptom management using medication and psychotherapy.

Despite the significant increase in PCPs’ pharmacotherapy or psychotherapy discussions and MI skills in the VW training group, participants viewed the synchronized virtual platform as less usable compared to those in the web-based training condition. This gap between the efficacy of the VW platform and its ease of use highlights the need for greater efforts in improving usability, particularly in terms of navigating avatars through virtual spaces using a standard personal computer and keyboard or mouse, as opposed to video game systems designed specifically for virtual world navigation.

### Limitations

This study’s findings are limited due to the attrition rate (approximately 50% in each group) from randomization to training as well as the small sample size. Nevertheless, the rates of dropout among PCP participants were similar across the 2 conditions. Moreover, high rates of attrition are common in studies of frontline clinical providers [25]. This suggests that future trials should provide greater incentives, include less burdensome study assessments, and plan for potential attrition in sample size calculations.

### Conclusions

The asynchronous web-based PTSD training was not as effective as the VW immersive experience in teaching PCPs to use MI skills in assessing and managing patients with PTSD but was viewed as more usable among PCPs. Participants in both platforms demonstrated increased PTSD-related knowledge and self-confidence in assessing and treating PTSD.

Improving PCP knowledge of trauma and PTSD and their MI communication skills is important to better engage patients in treatment and can serve as a vital gateway to specialty mental health treatment, given the high utilization rate of health care among individuals with PTSD [26]. Nonetheless, prior research indicates that PCPs require training in MI and PTSD assessment to increase PCP competence and comfort. VW training in PTSD and MI shows promise but requires moderate facilitation and technical support. As computer technology improves, immersive and interactive VW educational interventions may become more feasible and useful, particularly in teaching clinical and communication skills.

**Acknowledgments**

This work was supported by Department of Defense (award W81XWH-15-C-0088). We thank Adam Batten, BA, for his assistance with data analysis and the research participants for their involvement in this project.

## Appendix

Multimedia Appendix 1 Posttraumatic stress disorder and motivational interviewing skills coding.

Multimedia Appendix 2 Participant report of training platform usability.

Multimedia Appendix 3 CONSORT-eHEALTH checklist (V 1.6.1).

## Abbreviations

CME: continuing medical education
MI: motivational interviewing
PCP: primary care provider
PTSD: posttraumatic stress disorder
SMART: specific, measurable, action-oriented, realistic, and timebound
SP: standardized patient
SUS: System Usability Scale
VA: Veterans Affairs
VW: Virtual Worlds
