# Supplementary material for: Virtual Worlds Technology to Enhance Training for Primary Care Providers in Assessment and Management of Posttraumatic Stress Disorder Using Motivational Interviewing: Pilot Randomized Controlled Trial
Source: JMIR Med Educ. 2023 Aug 28;9:e42862. doi: 10.2196/42862 (PMC10495852; doi:10.2196/42862)
Supplement: Multimedia Appendix 1 [file mededu_v9i1e42862_app1.docx]

| **Multimedia Appendix 1. PTSD and MI Skills Coding** | | |
| --- | --- | --- |
| **Domain** | **Description** | **Analysis** |
| **A. Shared Decision-Making and Patient Engagement** | | |
| Assessing Functionality and Quality of Life* | Measures extent to which the clinician actively engages the patient to assess functioning and quality of life, including impact of PTSD symptoms on patient’s overall quality of life. | This item was removed from analyses due to low inter-rater reliability. |
| Overcoming Stigma | Measures clinician’s attempts to address PTSD stigma including eliciting patient’s own concerns about stigma | These two items are collapsed into single item, *Overcoming Stigma & Shared Decision Making.* |
| Shared Decision Making | Use of evidence-based practices to engage and partner with patient:   - Seeks patient participation - Assesses patient values and preferences - Helps explore and compare options - Reaches a decision with patient |  |
| **B. PTSD Symptom Assessment and Management** | | |
| B1. Assessment of PTSD Symptoms | Clinician asks about:   - Past trauma - Intrusions - Avoidance - Negative cognitions and mood - Alterations in arousal and activity | These two items are collapsed into single item, *B1. PTSD Symptoms & Co-Occurring Conditions***.** |
| B1. Assessment of PTSD Co-Occurring Conditions | Clinician asks about symptoms of:   - Suicidality and mental health conditions - Substance abuse disorders - Neurocognitive problems - Physical health - Sleep problems |  |
| B2. Discussion of Pharmacotherapy | Clinician discusses medication options:   - Identifies and offers pharmacologic treatment for PTSD symptoms - Explains purpose of medications for PTSD symptoms - Reviews risks and side effects - Reviews effectiveness of combination of medications and psychotherapy - Describes non-pharmacological treatments | These two items are collapsed into single item*, B2. Discussion of Pharmacotherapy & Discussion of Psychotherapies.* |
| B2. Discussion of Evidence-Based Psychotherapies | Clinician discusses psychotherapy options and introduces and describes different evidence-based practices for PTSD |  |
| **C. Motivational Interviewing** | | |
| Partnership | Clinician actively fosters power-sharing and elicits patient’s contributions throughout interaction. | These two items are collapsed into single item*, C: MI: Partnership and Empathy.* |
| Empathy | Clinician demonstrates understanding of patient’s perspective and experience. |  |
