# Supplementary material for: Virtual Worlds Technology to Enhance Training for Primary Care Providers in Assessment and Management of Posttraumatic Stress Disorder Using Motivational Interviewing: Pilot Randomized Controlled Trial
Source: JMIR Med Educ. 2023 Aug 28;9:e42862. doi: 10.2196/42862 (PMC10495852; doi:10.2196/42862)
Supplement: Multimedia Appendix 2 [file mededu_v9i1e42862_app2.docx]

|  | Total  N=85  mean (SD) | Web Training  N=45  mean (SD) | | Virtual Worlds  N=40  mean (SD) | | P |
| --- | --- | --- | --- | --- | --- | --- |
| System Usability Scale (0 – 100)* | 72.8 (24.7) | 86.8 | (17.4) | 56.6 | (21.7) |  |
| I think that I would like to use this type of training program frequently. | 2.6 (1.2) | 3.1 | (1.0) | 2.1 | (1.2) | <.001 |
| I found the training program unnecessarily complex | 2.6 (1.3) | 3.4 | (0.9) | 1.8 | (1.2) | <.001 |
| I thought the training program was easy to use | 3.0 (1.1) | 3.7 | (0.6) | 2.2 | (1.1) | <.001 |
| I think that I would need the support of a technical person to be able to use this training program. | 3.1 (1.3) | 3.8 | (0.6) | 2.2 | (1.3) | <.001 |
| I found the various functions in this training program well integrated | 2.9 (1.1) | 3.2 | (1.1) | 2.4 | (1.1) | <.001 |
| I thought there was too much inconsistency in this training program | 3.2 (1.0) | 3.7 | (0.6) | 2.8 | (1.0) | .001 |
| I would imagine that most people would learn to use this training program very quickly. | 2.9 (1.1) | 3.6 | (0.6) | 2.2 | (1.0) | <.001 |
| I found the training program very cumbersome to use | 2.9 (1.3) | 3.6 | (0.8) | 2.2 | (1.3) | <.001 |
| I felt very confident using the training program | 3.2 (1.0) | 3.7 | (0.5) | 2.5 | (1.0) | <.001 |
| I needed to learn a lot of things before I could get going with this training program. | 3.1 (1.2) | 3.7 | (0.7) | 2.4 | (1.3) | <.001 |

**Multimedia Appendix 2. Participant Report of Training Platform Usability**

*Scales were transformed to 0 – 4 for scoring, with higher scores indicating greater usability for all questions including questions which were asked in the negative (2, 4, 6, 8, and 10).
